# Campanacci Grade III Giant Cell Tumors of Distal End Radius Treated With Wide Excision and Reconstruction: A Retrospective Case Series

**DOI:** 10.7759/cureus.27818

**Published:** 2022-08-09

**Authors:** Eppakayala Srikanth, Nageswara Rao Kancherla, Bodla Arvind, Maheshwar Lakkireddy, Nagesh Cherukuri, Shravan Peddamadyam, Deepak Kumar Maley

**Affiliations:** 1 Department of Orthopaedics, All India Institute of Medical Sciences, Bibinagar, Hyderabad, IND; 2 Department of Orthopaedics, Nizam's Institute of Medical Sciences, Hyderabad, IND

**Keywords:** fibular grafting, benign tumours of distal radius, ulnar translocation, distal radius, giant cell tumours

## Abstract

Introduction

Campanacci Grade III Giant Cell tumors of the distal radius are difficult to manage as they are associated with a high recurrence rate. Wide excision of the distal radius and reconstruction with an ipsilateral proximal fibula or ulnar translocation reduces the recurrence rate significantly and gives acceptable function to the hand and wrist.

Methods and materials

This was a retrospective study of eight patients with Campanacci grade III giant cell tumors of distal radius treated with wide excision of distal radius followed by reconstruction at our institute. Four cases were operated on with ulnar translocation and four cases were operated on with ipsilateral proximal fibula grafting after wide excision of the distal radius. Patients were studied for the Musculoskeletal Tumor Society (MSTS) score and visual analogue scale (VAS) score for pain at one year, recurrence, and complications.

Results

The mean MSTS score of the total series was 24.75 ± 1.6. The mean VAS score for the total series was 1.62 ± 0.4. Of the eight cases, two cases had a recurrence, one patient had persistent wrist paint, and two patients had wrist subluxation.

Conclusion

Wide excision of the distal radius followed by reconstruction with a proximal fibula or ulnar translocation is a good option to avoid repeated surgeries in patients with Campanacci grade III giant cell tumors of the distal radius and achieve acceptable functional results for the wrist and hand.

## Introduction

Giant cell tumors (GCTs) are locally aggressive benign bone tumors. They are commonly seen in epi-metaphyseal regions of long bones. The distal end of the radius is the third most commonly involved region after the distal femur and proximal tibia [1]. Of GCTs, 70% occur in the age group of 20-45 years [2]. Campanacci grade I and grade II GCTs of the distal radius are confined to bone and do not involve the surrounding soft tissues. They respond well to intralesional excision with extended curettage followed by filling the defect with autogenous or allogenous bone graft or bone graft substitute.

Campanacci grade III GCTs involve the surrounding soft tissues following a break in the cortex. Recurrence following extended curettage and bone grafting in Campanacci grade III lesions is high. According to a meta-analysis done by Liu et al., there was a five-fold increase in recurrence with extended curettage and bone grafting compared to en bloc excision followed by reconstruction [3]. Available reconstructive options after en bloc excision of the tumor include arthrodesis with vascularized or nonvascularized autografts like proximal fibula, distal ulna, iliac crest, or osteoarticular allograft transplantation or arthroplasty with a custom made prosthesis [4-9].

The present study evaluates the functional outcome of eight patients with Campanacci grade III GCTs of distal radius treated with wide excision and reconstruction.

## Materials and methods

This was a retrospective study conducted by using medical records of eight patients diagnosed with Campanacci grade III GCTs of the distal end of radius treated with wide excision and reconstruction with ulnar translocation or nonvascularized proximal fibular autograft at Nizam's Institute of Medical Sciences, Hyderabad, India, between January 2016 and December 2021.

Subject selection

Inclusion criteria were age of the patients between 20 years and 50 years and patients with histopathologically confirmed Campanacci grade III GCTs of the distal radius. Exclusion criteria were age<20 years and >50 years, patients with Campanacci grade I and grade II GCTs of the distal radius, patients with GCTs at other skeletal and extraskeletal sites, and recurrent GCTs of the distal radius.

Case series

**Figure 1:**
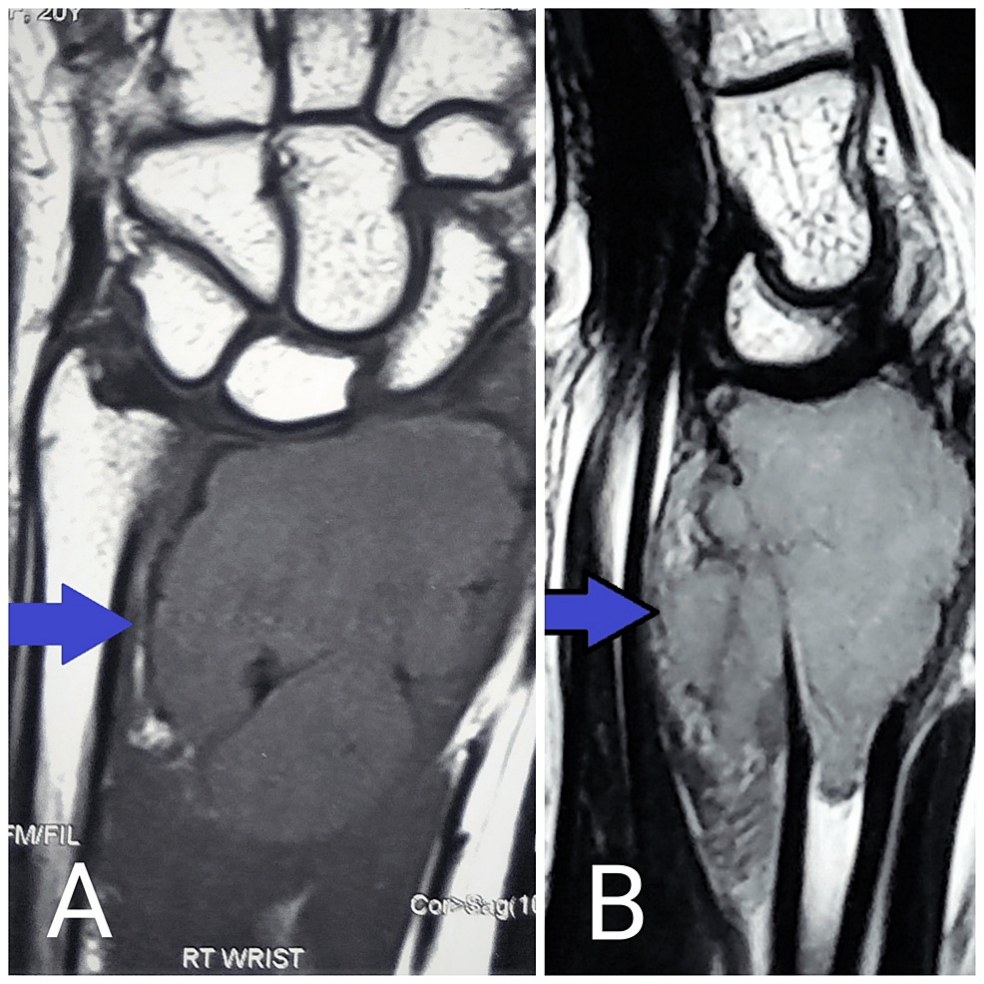
MRI of wrist showing cortical breech, medial and volar soft tissue involvement by tumor

All patients presented with pain and swelling at the wrist joint. Patients were evaluated with radiographs and MRI. MRI was done to determine the presence of pathological fracture and the soft tissue extent of the tumor (Figure 1).

**Figure 2:**
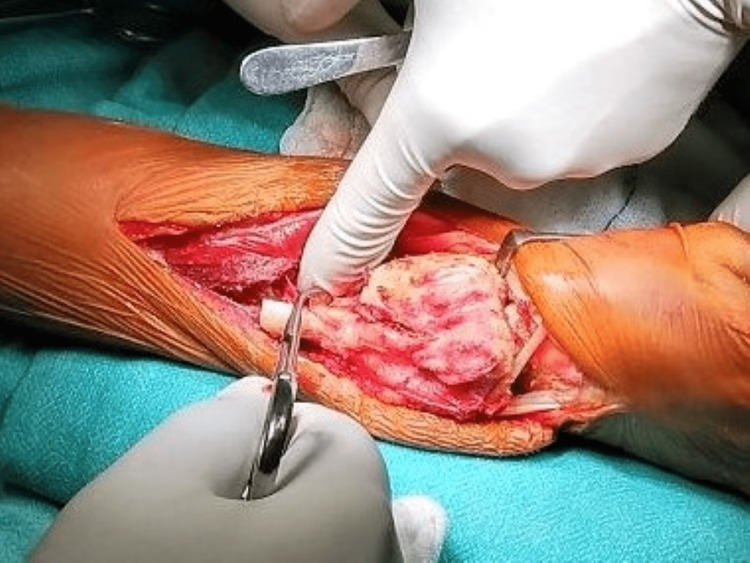
Intraoperative picture showing wide/en bloc excision of the tumor

Patients were then planned for core needle biopsy. After histopathological confirmation of GCT, patients were subjected to definitive surgery. All patients were operated on under general anesthesia and tourniquet control. In all the cases, the distal radius was exposed by dorsal approach and the tumor was excised en bloc taking a clear margin of 2 cms (Figure 2).

Reconstruction was done by ulnar translocation and arthrodesis or ipsilateral proximal fibular autograft. In this case series, four patients were treated with ulnar translocation and arthrodesis and another four patients were treated with ipsilateral proximal fibula autograft and arthroplasty following wide/en bloc excision of distal radius. All the subjects were assessed with MSTS score and VAS score for pain at one year, recurrence, and complications.

MSTS functional score system measures outcomes in six categories including pain, function, and emotional acceptance. For upper extremity weight lifting, hand position and lifting ability were recorded. Each parameter is scored 0 to 5 and combined for a possible total score of 30. Intermediate values of 2 or 4 were assigned, based on the examiner's judgment, when achievement or performance falls between the specified values (Table 1).

**Table 1:** MSTS score for upper limb MSTS: Musculoskeletal Tumor Society

Score	Pain	Function	Emotional	Hand Positioning	Manual Dexterity	Lifting Ability
5	No pain	No restriction	Enthused	Unlimited	Unlimited	Normal load
4	Intermediate	Intermediate	Intermediate	Intermediate	Intermediate	Intermediate
3	Modest/nondisabling	Recreational restriction	Satisfied	Not above shoulder or no pronation/supination	Loss of fine movements	Limited
2	Intermediate	Intermediate	Intermediate	Intermediate	Intermediate	Intermediate
1	Moderate/disabling	Partial restriction	Accepts	Not above waist	Cannot pinch	Helping only
0	Severe disabling	Total restriction	Dislikes	None	Cannot grasp	Cannot help

In lifting ability, "Limited" indicates limitations in independent lifting, "Helping" means the patient cannot lift the upper limb independently but is useful in assisting the contralateral upper limb in doing activities. A score of 23 or greater is considered an excellent result, a score of 15 to 22 points a good result, a score of 8 to 14 points a fair result, and a score less than 8 a poor result [10].

Pain relief is evaluated by VAS that grades pain from 0-10, where 0 is no pain and 10 is the worst pain. VAS score was recorded at every follow-up [11].

## Results

**Figure 3:**
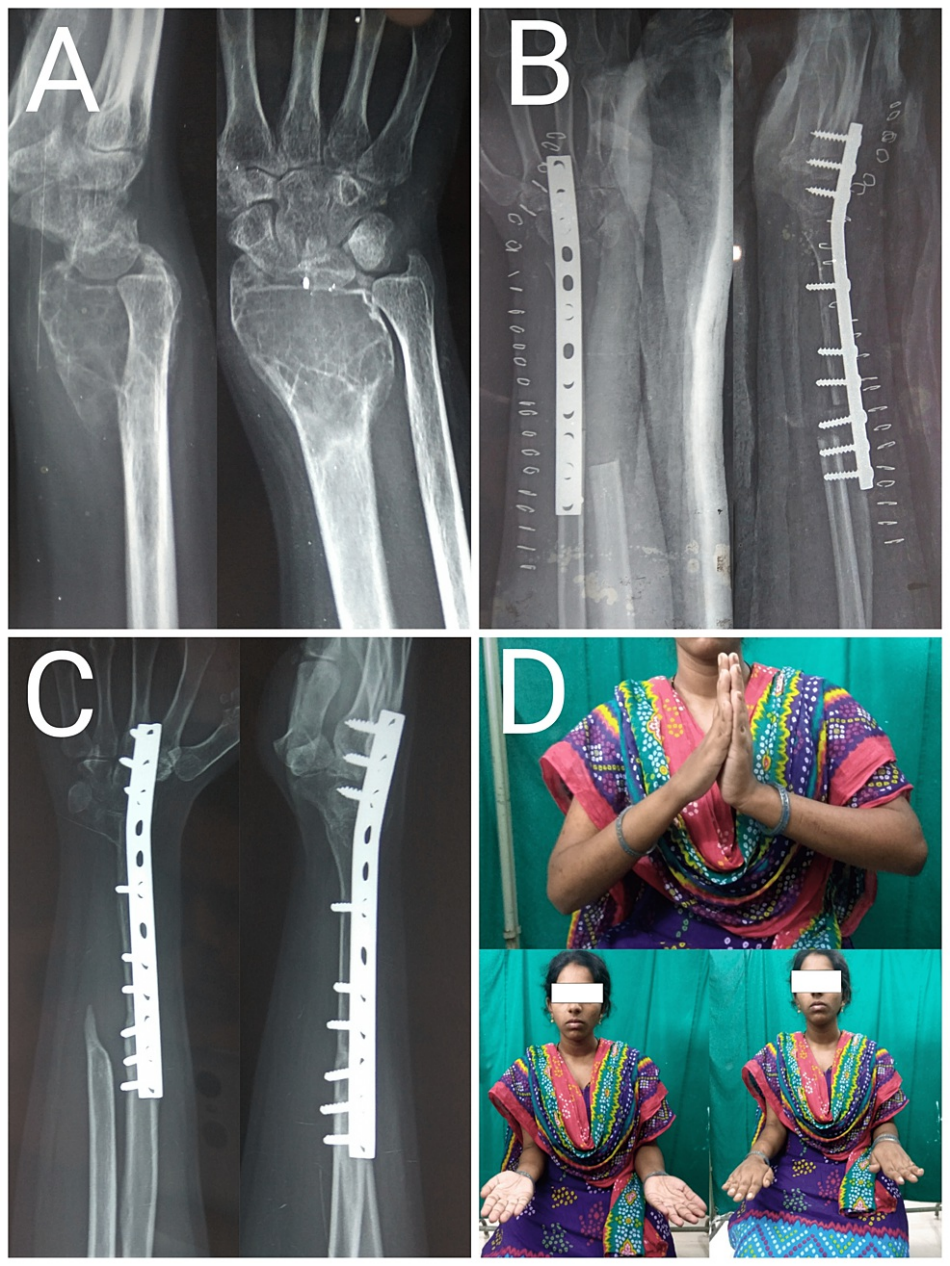
Case images of a 20-year-old female patient with Campanacci grade III giant cell tumor of right distal radius (A) Preoperative radiograph showing giant cell tumor of radius; (B) Immediate postoperative radiograph showing wide excision and ulnar translocation; (C) One-year post-operative radiograph showing union at ulnoradial and ulnocarpal junction; (D) Good forearm rotations and acceptable wrist and hand function

**Figure 4:**
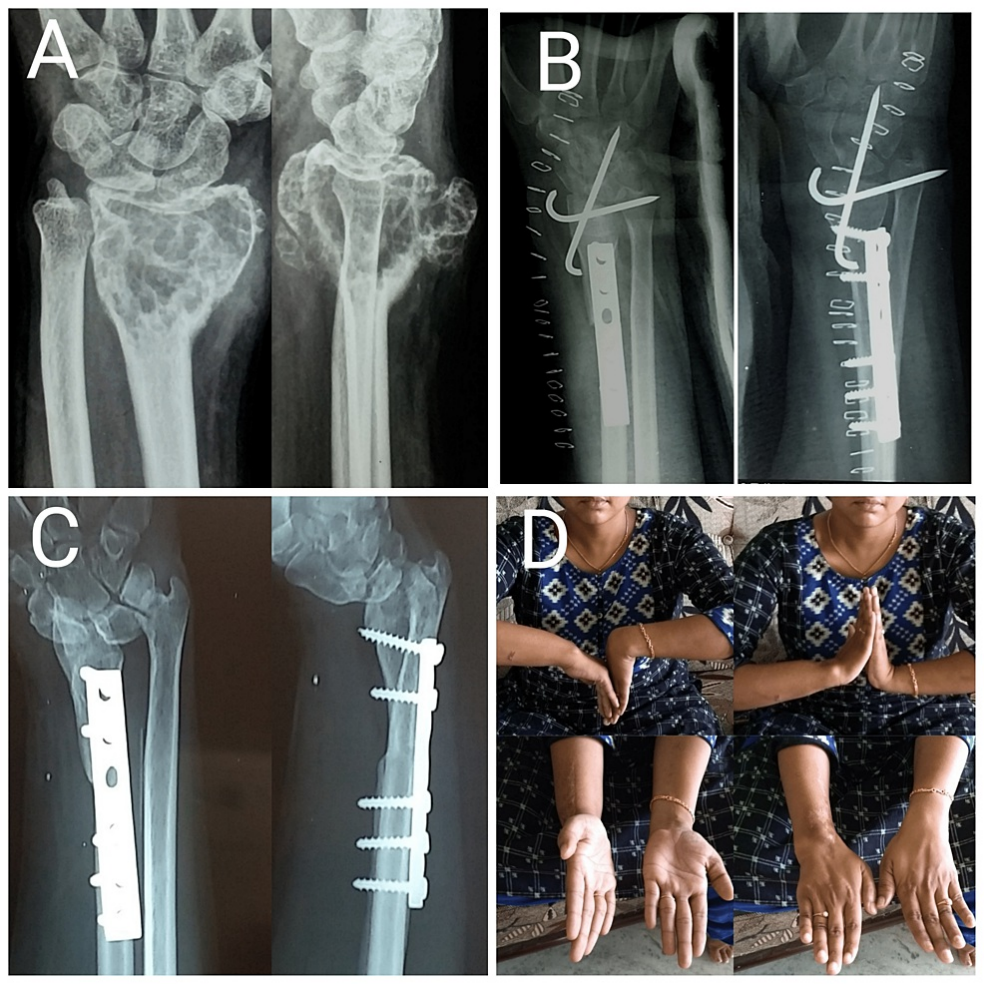
Case images of a 23-year-old female patient with Campanacci grade III giant cell tumor of right distal radius (A) Preoperative radiograph showing giant cell tumor of distal radius; (B) Immediate postoperative radiograph showing wide excision and ipsilateral proximal fibular autograft; (C) One-year post-operative radiograph showing union at fibuloradial junction with subluxation at wrist joint; (D) Reduced forearm rotations but good wrist and hand function after fibular autograft.

The mean age of the study group was 26.7 ± 2.6 years with a range of 20-44 years. Of the eight patients, four were males and four were females. All the patients were followed for at least 14 months, with a mean follow-up of 22.25 ± 2.4 months (range:14-36 months). The mean MSTS score was 27.25 ± 1.1 for the ulnar translocation group compared to 22.5 ± 2.5 for the fibular grafting group. The mean MSTS score of the total series was 24.75 ± 1.6. The mean VAS score was 1.25 ± 0.6 for the ulnar translocation group whereas the VAS score for the fibular grafting group was 2 ± 0.7. The mean VAS score for the total series was 1.62 ± 0.4. Of the four cases operated with fibular grafting, two cases had a recurrence, which was operated with resection of the tumor followed by ulnar centralization. One patient operated with wide excision and ulnar translocation had persistent wrist pain. Of the four patients operated with wide excision and ipsilateral proximal fibular autograft, two patients had wrist subluxation. Summary of the eight patients included in the case series is shown in Table 2. Case examples of ulnar translocation and fibular grafting are shown in Figures 3, 4.

**Table 2:** Summary of eight cases included in the study MSTS: Musculoskeletal Tumor Society; VAS: visual analogue scale

S. no.	Age (years)/Sex	Site of Involvement	Treatment Modality	Follow-up (months)	MSTS Score	VAS Score	Recurrence	Complications
1	28/F	Right distal radius	Wide/En bloc Excision +ulnar translocation	22	28	1	No	Nil
2	20/F	Right distal radius	Wide/En bloc Excision +ulnar translocation	36	29	0	No	Nil
3	26/M	Right distal radius	Wide/En bloc Excision +ulnar translocation	18	24	3	No	Persistent wrist pain
4	24/F	Left distal radius	Wide/En bloc Excision +ulnar translocation	28	28	1	No	Nil
5	24/F	Right distal radius	Wide/En bloc Excision and Ipsilateral fibula grafting	18	16	4	Yes	Wrist subluxation
6	44/M	Right distal radius	Wide/En bloc Excision and Ipsilateral fibula grafting	20	20	2	Yes	Wrist subluxation
7	26/M	Left distal radius	Wide/En bloc Excision and Ipsilateral fibula grafting	22	27	1	No	Nil
8	22/M	Left distal radius	Wide/En bloc Excision and Ipsilateral fibula grafting	14	26	1	No	Nil

## Discussion

Management of Campanacci grade III GCTs of the distal radius is challenging due to the aggressive clinical behavior of the tumor and difficulty in reconstructing the radiocarpal joint owing to its complex anatomy [12]. Owing to the high risk of recurrence with extended curettage, wide excision of the distal radius followed by reconstruction with a suitable graft or prosthesis is preferred [13].

The morphology of the proximal fibula is similar to the distal radius and helps to form the wrist joint that allows an acceptable range of motion. The use of nonvascularized proximal fibula autograft to reconstruct the defect and wrist joint was described by Mays et al. [14]. This method has low donor site morbidity and relatively fewer major complications. Wrist subluxation, delayed union, nonunion, graft resorption, laxity of the lateral ligament at the knee, and peroneal nerve palsy are some of the known complications [5]. Wrist subluxation is the most common complication associated with proximal fibular grafting but it does not appear to reduce the functional outcome as seen in other studies [15-18]. Advantages of nonvascularized proximal fibula autograft is preserved movements at the wrist and cosmetically better-looking limb. Wide/en bloc excision and arthrodesis with ulnar translocation is another viable option that preserves forearm rotations and gives good grip strength. Disadvantages are complete loss of movement at the wrist and narrowing of the wrist and distal forearm giving an hourglass appearance to the upper limb.

In this study, we report eight cases of Campanacci grade III GCTs of distal radius managed with wide excision and reconstruction with an ulnar translocation or ipsilateral proximal fibular graft. The mean age in this study was 26.7 years, which was comparable to the study of Salunke et al. (29 years) [19]. The mean MSTS score in this study was 27.25 for the ulnar translocation group, which is slightly higher compared to the study reported by Salunke et al. and Puri et al. [19,20]. This may be due to the small sample size of this study. The mean MSTS score was 22.25 for the fibular grafting group and is significantly lower compared to the study of Saini et al. at 27.3 (91%) [16]. This was because of two recurrences in the fibular grafting group that affected MSTS score significantly. All the patients in this study had good pain relief with both the procedures with a mean VAS score of 1.62. In this study, two out of eight patients had local recurrence (25%). This was due to the aggressive nature of GCTs and excessive soft tissue involvement precluding complete tumor clearance despite the best possible surgical practices. These two patients were from the fibular grafting group and were reoperated with excision of the tumor and ulnar centralization described by Hey Groves [21].

Three patients had postoperative complications: one had persistent wrist pain and two patients had wrist subluxation. Wrist subluxation did not reduce the functional outcome of the procedure as both the patients were functionally doing well. There were no nonunions in both groups. There was no lateral ligament laxity or peroneal nerve palsy in fibular grafting cases. Review of the literature of previous studies with more than 10 cases is presented in Table 3.

**Table 3:** Review of literature of case series (with more than 10 cases) of distal radius giant cell tumor treated with en bloc excision and reconstruction with ulnar translocation or ipsilateral proximal fibular grafting

Author, Year	No. of Cases	Treatment Method	Results	Recurrence	Complications
Puri et al. [20], 2010	12	Enbloc excision+ulnar translocation	Excellent -11, Good-1	3 cases	Superficial flap necrosis-3, radioulnar synostosis-1, post-traumatic fracture-1
Saini et al. [16], 2011	12	Enbloc excision+autogenous fibular grafting	Excellent -5, Good-4, Satisfactory-3	1 case	Subluxation-3, non union-2
Salunke et al. [19], 2017	25	Enbloc excision+ulnar translocation	Excellent- 24, Good-1	1 case	Graft fracture- 2 cases
Lackman et al. [22], 1987	12	Enbloc excision+autogenous fibular grafting	Excellent -6, Good-4, Fair-2	1 case	Nonunion-2, graft fracture-3, subluxation-1
Saikia et al. [15], 2010	24	Enbloc excision+autogenous fibular grafting	Excellent -6, Good-14, Fair-4	1 case	Subluxation-10, infection-1, graft fracture-1
Aithal et al. [23], 2003	30	Enbloc excision+autogenous fibular grafting	Good-11, Fair-2, Poor-2 (excluding recurrences)	10 cases	Nonunion-3, infection-1, subluxation-3

In this study, we have taken only Campanacci grade III GCTs of distal radius as the study group, which may be considered a focused analysis. However, a comparison of ulnar translocation, fibular grafting, and distal radius prosthesis in a single study would be more useful in determining a standard treatment approach for Campanacci grade III GCTs of the distal radius. Small sample size, lack of control group, short follow-up, and retrospective nature of study are the limitations of this study.

## Conclusions

Campanacci grade III GCTs of distal radius carry a high risk of recurrence following extended curettage and bone grafting. Wide/en bloc excision of distal radius followed by reconstruction with proximal fibular autograft or ulnar translocation is a reasonably good option to avoid repeated surgeries and achieve acceptable functional results for the wrist and hand. Both of these procedures are easy, do not need any microvascular procedures, and have fewer complications.
